# Neurodegeneration-associated FUS is a novel regulator of circadian gene expression

**DOI:** 10.1186/s40035-018-0131-y

**Published:** 2018-10-12

**Authors:** Xin Jiang, Tao Zhang, Haifang Wang, Tao Wang, Meiling Qin, Puhua Bao, Ruiqi Wang, Yuwei Liu, Hung-Chun Chang, Jun Yan, Jin Xu

**Affiliations:** 1 Institute of Neuroscience, State Key Laboratory of Neuroscience, CAS Key laboratory of Primate Neurobiology, Shanghai Institutes for Biological Sciences, Chinese Academy of Sciences, New Life Science Bldg, 320 Yue Yang Road, Shanghai, 200031 China; 2 University of Chinese Academy of Sciences, Shanghai, 200031 China

**Keywords:** FUS, ALS, FTD, Circadian rhythm, Gene regulation

## Abstract

**Background:**

Circadian rhythms are oscillating physiological and behavioral changes governed by an internal molecular clock, and dysfunctions in circadian rhythms have been associated with ageing and various neurodegenerative diseases. However, the evidence directly connecting the neurodegeneration-associated proteins to circadian control at the molecular level remains sparse.

**Methods:**

Using meta-analysis, synchronized animals and cell lines, cells and tissues from FUS R521C knock-in rats, we examined the role of FUS in circadian gene expression regulation.

**Results:**

We found that FUS, an oscillating expressed nuclear protein implicated in the pathogenesis of amyotrophic lateral sclerosis (ALS) and frontotemporal dementia (FTD), exerted a novel feedback route to regulate circadian gene expression. *Nr1d1-*encoded core circadian protein REV-ERBα bound the *Fus* promoter and regulated the expression of *Fus*. Meanwhile, FUS was in the same complex as PER/CRY, and repressed the expression of E box-containing core circadian genes, such as *Per2*, by mediating the promoter occupancy of PSF-HDAC1. Remarkably, a common pathogenic mutant FUS (R521C) showed increased binding to PSF, and caused decreased expression of *Per2*.

**Conclusions:**

Therefore, we have demonstrated FUS as a modulator of circadian gene expression, and provided novel mechanistic insights into the mutual influence between circadian control and neurodegeneration-associated proteins.

**Electronic supplementary material:**

The online version of this article (10.1186/s40035-018-0131-y) contains supplementary material, which is available to authorized users.

## Background

Circadian rhythms are oscillating physiological and behavioral changes governed by an internal molecular clock, and rely on two transcriptional feedback loops to regulate the expression of various core circadian genes [1–3]. BMAL1 and CLOCK transcriptionally activate E-box-containing genes, including *Per*, *Cry* and *Nr1d1/2*. When the protein products of *Per*, *Cry* and *Nr1d1/2* build up, *Nr1d1/2*-encoded REV-ERBα/β can repress the transcription of *Bmal1* by binding to retinoic acid–related orphan receptor response (ROR) elements in the *Bmal1* promoter [4, 5], and PER and CRY proteins can join a mega-transcriptional repressor complex with BMAL1 and CLOCK to shut down the expression of E-box-containing genes [6–11]. The strength of these two major transcriptional feedback loops can be further fine-tuned by additional transcriptional co-regulators [12–14]. For example, RNA/DNA-binding protein PSF (also known as splicing factor proline and glutamine rich) could act as a transcriptional co-repressor by recruiting SIN3A-HDAC1 to rhythmically deacetylate *Per1* promoter and repress the transcription of *Per1*[12].

There is a plethora of evidence connecting circadian rhythm dysfunction to neurodegeneration [15, 16]. However, except for Ataxin-2 in *Drosophila* circadian locomotor behavior regulation [17, 18], there is little evidence mechanistically links neurodegenerative disease-associated proteins to the regulation of circadian clock. FUS is a nuclear protein implicated in the pathogenesis of ALS and FTD [19–21]. Mutations in FUS cause early onset of ALS, and the accumulation of FUS is a common feature in FTD neuropathology [21]. Furthermore, sleep disorders are known to affect some ALS and FTD patients [22–24]. *Fus* is suggested as a potential circadian regulated gene with oscillating mRNA expression in mouse liver, prefrontal cortex, skeletal muscle and other tissues [25, 26]; however, its regulation by circadian clock has never been characterized and its role in circadian regulation is unknown. In this study, we found that FUS is not only transcriptionally regulated by REV-ERBα, but also modulates the expression of *Per* and *Cry*. Therefore, our study provides novel insights into mutual regulation between circadian control and neurodegeneration-related proteins.

## Methods

### Animals

All animal works were performed in accordance with the regulations by the Animal Care and Use Committee of the Institute of Neuroscience, Shanghai Institutes for Biological Sciences.

The detailed procedures for the establishment of the FUS-R521C knock-in rats via CRISPR/Cas9, and the characterization of the animals were described elsewhere (T.Z. et al, *Neurobiology of Aging,* in press; Additional file 1). Briefly, Cas9 mRNA, single guide RNA (sgRNA) targeting the C-terminus of rat *Fus* gene, and donor DNA were injected into the cytoplasm of zygotes of the Sprague Dawley rats (Additional file 1: Figure S1). The sequence for sgRNA and donor DNA are: TGAGCACAGACAGGATCGCA (sgRNA), TTAATCTAACAAATAATTTTTTCTTTCAGG GGTGAGCACAGACAGGATTGCAGGGAGAGGCCATATTAGCCTGACTCCTGAAGTTCTGGAACAGCTCTTC (donor DNA). The presence of inserted mutation was determined by PCR followed by sequencing. The potential off-target effects in F0 (founder) rats were estimated using Cas-OFFinder [27] and assessed by PCR-sequencing. Multiple rounds of breeding were carried out to eliminate off potential off target effects. The *Nr1d1* knock-out mice were described previously [5].

### Cell culture, transfection and synchronization

Rat embryonic fibroblasts (REFs) were collected from E13.5-15.5 embryos using pregnant rats from heterozygous R521C FUS knock-in rats mating pairs. Mouse embryonic fibroblasts (MEFs) were collected from E13.5-E15.5 embryos using pregnant mice from heterozygous *Nr1d1* knock-out mice mating pairs. REFs, MEFs, HEK293T cells (ATCC) and Neuro-2a (ATCC) cells were cultured at 37 °C in 5% CO_2_ in DMEM (for REFs and HEK293T cells, Gibco c11965) or DMEM/F-12 (for Neuro-2a cells, Gibco c11330) medium, supplemented with 10% fetal bovine serum (Gibco, 10099) and antibiotics (Penicillin and streptomycin, HyClone, SV30010).

Cells were transfected using Lipofectamine 2000 reagent (Invitrogen, 11668) for over-expression or Lipofectamine RNAiMAX (Invitrogen, 13778) for gene silencing. Control plasmids (GFP) or scrambled siRNA (Ctrl) were used to make sure equal amount of the constructs were transfected in each condition. Typically, cells were harvested 48 hrs for over-expression and 72 hrs for RNAi silencing after transfection.

Neuro-2a cell were synchronized as described previously [13]. Briefly, 48 hrs after siRNA silencing or 24 hrs after over-expression, the cultural medium for transfected cells were changed to 50% horse serum (Gibco, 16050)-50% DMEM/F12 for 2 hrs followed by culturing in 1%-FBS-containing DMEM for 22 hrs before harvesting. For REFs synchronization, 48 hrs after transfection, cells were cultured in DMEM (with 1% FBS and antibiotics) containing 10μM forskolin (Sigma, F3917) for 2 hrs, followed by culturing in the low serum condition until the end of the experiment (DMEM with 1% FBS) as described [28]. The synchronization of MEFs was as described [29, 30]. MEFs were treated with 100nM Dexamethasone for 1 hr when cells reach confluence, then the cells were cultured in the low serum condition (DMEM with 1% FBS) for 36 hr followed by harvesting.

### Circadian tissue collection

**Fig. 1:**
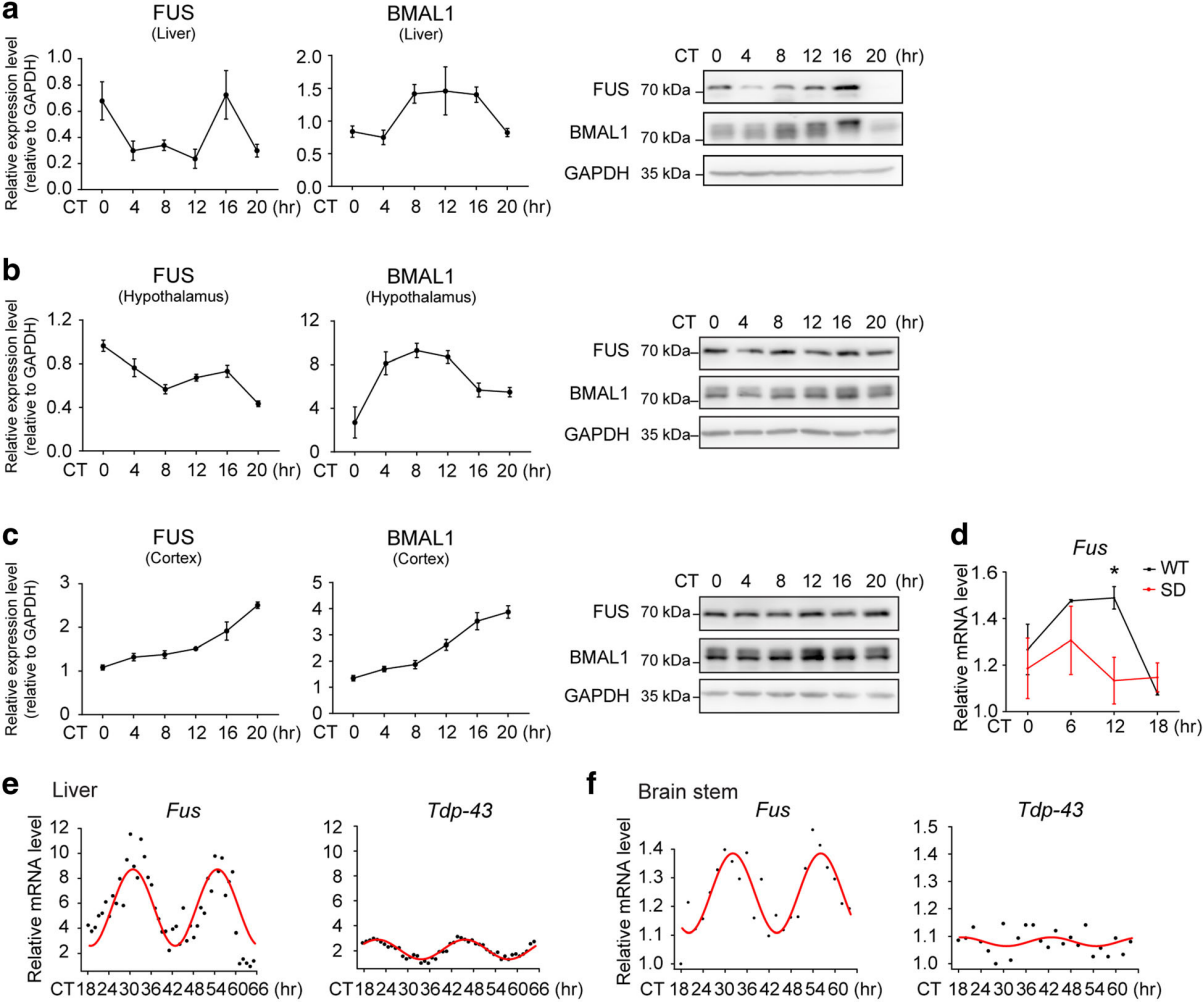
*Fus* is a circadian regulated gene. **a-c**. Western blot showing the protein expression of FUS in the liver (**a**), hypothalamus (**b**) and cortex (**c**) of free-running wild-type mice (CT: circadian time), the quantification was shown on the left (mean ± s.e.m.; *N* = 5 mice were sacrificed at each time point). **d** mRNA expression level of *Fus* in the whole brain of sleep-deprived mice using microarray datasets [31] (mean ± s.e.m.; *N* = 3 experiments, *t*-test, *:*P*≤0.05). (**e**, **f**) mRNA expression level of *Fus* and *Tdp-43* in the liver [37] (**e**) and brain stem [38] (**f**) of mouse. The lowest value for the dataset in each graph was set as 1 to determine relative fold change

For Fig. 1a-c, 7-week-old male wild-type C57BL/6 mice (SLAC Laboratory Animal, Shanghai) were maintained in a light-tight, ventilated, temperature (22 °C) and humidity (60%)-controlled animal facility with free access to food and water. The lighting schedule was 12 hr light:12 hr dark (lights on at 7 a.m.). To measure the endogenous circadian gene oscillation, entrained mice were then released to a constant low irradiance light condition (~30 Lux, measured at the bottom of cage) for one week. Starting at CT-8 (circadian time), five mice were sacrificed every 4 hrs, for 24 hrs. Tissues were quickly dissected and frozen immediately in solid carbon dioxide for further analysis.

For Fig. 1d, the detailed method for sleep deprivation (SD) was as described [31]. Briefly, SD was initiated at ZT-0 hr (Zeitgeber time), ZT-6 hr, ZT-12 hr and ZT-18 hr by gentle-handing method for 5.5 hrs, mice were then sacrificed within the next 30 min.

**Fig. 2:**
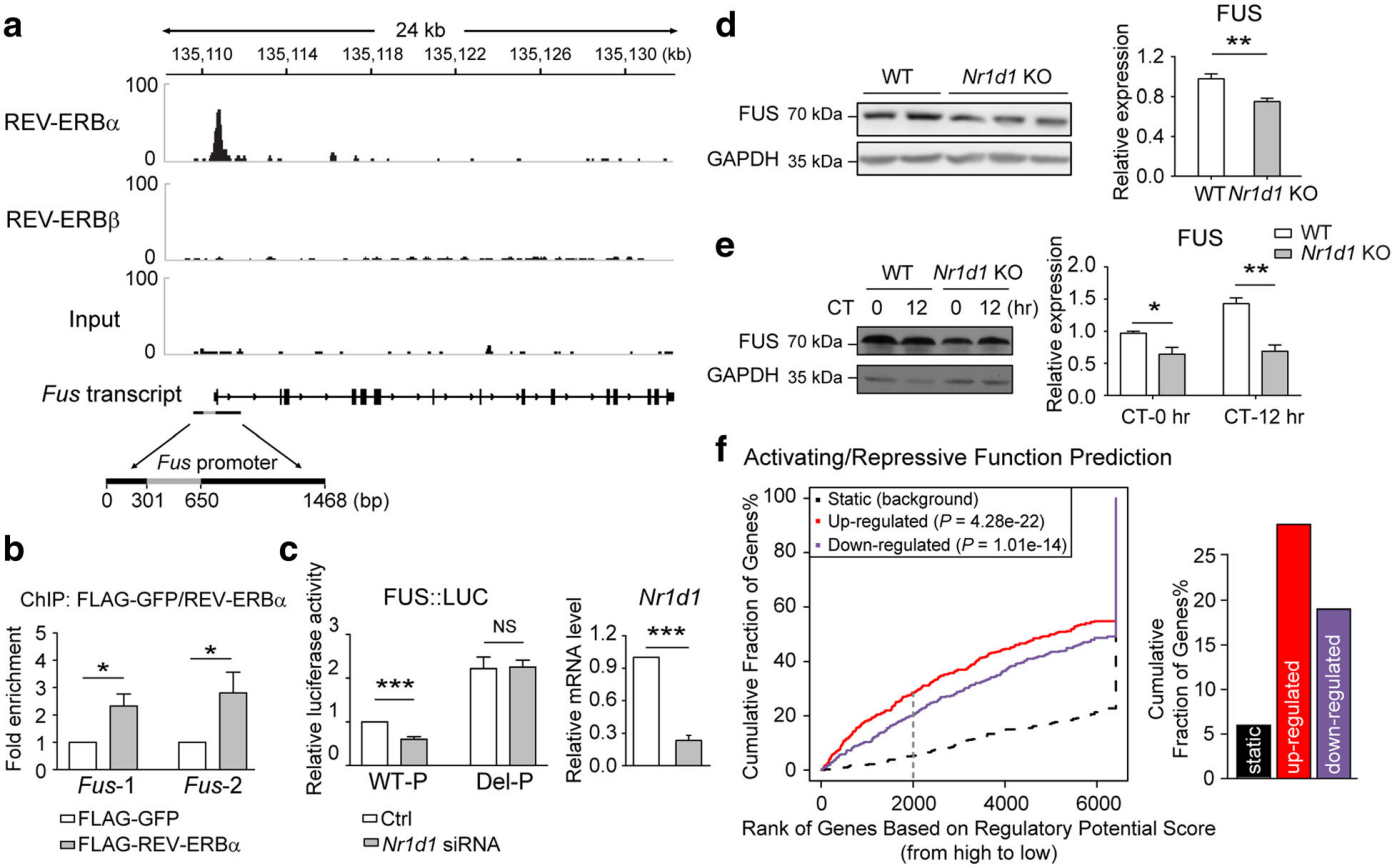
REV-ERBα activates the circadian expression of FUS. **a** ChIP-seq analysis showing REV-ERBα binding signals on the *Fus* promoter. The black bar below indicates the *Fus* promoter region (WT-P in Fig. 2**c**) used in the *Fus* promoter-luciferase construct, while the grey area in the middle of black bar indicates the region harboring the REV-ERBα-binding site based on ChIP-seq data [32]. **b** ChIP-qPCR showing the binding of FLAG-REV-ERBα to the *Fus* promoter in Neuro-2a cells (*Fus*-1 and *Fus*-2 are two pairs of primers specific for regions located in the predicted REV-ERBα binding sites in Fig. 2**a**; FLAG-GFP was used as the control; mean ± s.e.m.; *N* = 4 experiments; *t*-test; *:*P*≤0.05). **c** Luciferase activity of the intact (WT-P) and REV-ERBα-binding site deleted (Del-P) *Fus* promoter-luciferase constructs in Neuro-2a cells after siRNA silencing (mean ± s.e.m.; *N* = 4 experiments; *t*-test; ***:*P*≤0.001). The right panel showed the knock-down efficiency of *Nr1d1*-targeting siRNA (mean ± s.e.m.; *N* = 4 experiments; *t*-test; ***:*P*≤0.001; Ctrl represents scrambled control siRNA; NS: non-significant). **d** FUS expression level in synchronized wild-type or *Nr1d1* knock-out (KO) MEFs. Quantification result was shown in the bar graph (mean ± s.e.m.; three lines of wild-type MEFs and four lines of *Nr1d1* KO MEFs were generated from two pregnant *Nr1d1* heterozygous mice, *t*-test; **: *P*≤0.01). **e** FUS expression level in the liver of free-running wild-type and *Nr1d1* knock-out mouse at indicated time point, quantification result was shown in the right bar graph (*N* = 3 pairs of littermates for CT-0 hr and *N* = 2 pairs for CT-12 hr; mean ± s.e.m.; two-way ANOVA with Sidak's multiple comparison test, *:*P*≤0.05, **:*P*≤0.01). **f** Activating/repressive functional prediction analysis based on the published REV-ERBα ChIP-seq data [32] and transcriptional profile in *Nr1d1* knock-out mice [53]. Genes are cumulated by the rank on the basis of the regulatory potential score from high to low according REV-ERBα ChIP-seq data (x-axis). The red and purple lines represent the percentage of up-regulated (UP) or down-regulated (DOWN) genes that harbor REV-ERBα binding sites from *Nr1d1* knock-out microarray data, respectively. The black dashed line indicates the non-differentially (NON) expressed genes among REV-ERBα-binding genes. *P* values that represent the significance of the UP or DOWN group distributions are compared with the NON group by the Kolmogorov-Smirnov test. The right panel is an example showing the fraction of up-regulated (red) or down-regulated genes (purple) that contain REV-ERBα binding sites when the top 2,000 peaks from the ChIP-seq data were included (gray dash line in the left panel). The cumulative fractions of genes that are down-regulated in *Nr1d1* knock-out mice indicate that REV-ERBα could also act as an activator

For Fig. 2d, 3.5-month-old *Nr1d1*-knockout mice and littermate wild-type control mice were entrained in the 12 hr:12 hr light:dark condition for more than a week then released to constant dark condition for five days. Three pairs of littermate mice were sacrificed at CT-0 hr and two pairs of mice were sacrificed at CT-12 hr under the dim red light. Tissues were quickly dissected and frozen immediately in solid carbon dioxide for further analysis.

### Dual-luciferase assay

Firefly luciferase reporter constructs directed by the intact mouse *Fus* promoter (~1.5kb upstream) or the promoter with predicted REV-ERBα-binding site (350 bp [32]) deleted were PCR amplified from mouse genomic DNA and cloned into firefly luciferase reporter vector (pGL3-Basic Vector, Promega, E1751). Firefly luciferase reporter constructs directed by mouse *Per2* promoter (1.7 kb) [33] was a gift from Dr. Hung-Chun Chang’s lab. Cells were transfected with firefly luciferase reporter with renilla luciferase reporter (pRL-SV40 Vector, Promega, E2231) as internal control. According to the technical manual of Dual-Luciiferase Reporter Assay System (Promega, E1910), 48 hrs after transfection, cells were eventually lysed with Passive Lysis Buffer. Then, transferred 20μL of cell lysate into 100μL of LARII reagent and the luminescence of firefly luciferase reporter was read by the tube luminometer (Titertek Berthold). Next, added 100μL of Stop & Glo reagent and put the tube back to the luminometer again and read the luminescence for internal renilla luciferase control. For each experiment, samples were analyzed in duplicates. Sequences of siRNAs were listed in Additional file 2: Table S1.

### Plasmids, siRNA, and Antibodies

FLAG-mouse REV-ERBα and FLAG-PSF, FUS, R521C and 1-360 FUS were generated by PCR cloning. The primary antibodies used are: rabbit anti-PSF (Sigma, PLA0181; WB: 1:2,000, IP: 1:100; ChIP: 1:100), mouse anti-FUS (Santa Cruz, sc-4H11; WB: 1:1,000), mouse anti-DYKDDDDK-Tag (FLAG) (Abmart, M2008; WB:1:5,000), mouse anti-DYKDDDDK-Tag conjugated protein A/G beads (Abmart, M200018; IP and ChIP: 35uL beads per each assay), mouse anti-PSF antibody (Sigma, P2860; WB: 1:1,000), rabbit anti-CLOCK (Cell Signaling Technology, D45B10; WB:1:3,000), rabbit anti-BMAL1 (CST, D2L7G; WB: 1:3,000), rabbit anti-PER2 (Abcam, ab179813; WB: 1:1,000), mouse anti-ACTIN (Abmart, M20010; WB: 1:5,000), rabbit anti HDAC1 (Abcam, ab7028; ChIP: 1:150; WB: 1:2,000), mouse anti-TUBULIN (Abmart, T40103; WB: 1:5,000), mouse-anti GAPDH (Proteintech, 60004-1-Ig; WB: 1:10,000). Primer sequences are included in Additional file 2: Table S1.

### Immunoblotting, immunoprecipitation and chromatin-immunoprecipitation

The procedure for western blotting was as described previously [34]. Briefly, animal tissue samples or cultured cells were lysed in RIPA buffer (150 mM NaCl, 50 mM Tris buffer (pH=8.0), 1% NP-40, 1% deoxycholate and 0.1% SDS) with protease inhibitors (Roche, 5892970001). After centrifugation at 12,000 rpm for 15 min at 4 °C, the concentration of soluble fraction was measured by BCA Protein Assay Kit (Tiangen) and the soluble fraction was boiling in 5× loading buffer at 100 °C for 15 min. Around 40 μg of protein was loaded per lane for western blotting.

The procedure for immunoprecipitation was described previously with minor modification [35]. Briefly, transfected HEK293T cells (10 cm-plate, 48 hrs after transfection) or the whole brain of rats were lysed in NP-40 buffer (150mM NaCl, 1% NP-40, 50 mM Tris buffer (pH=8.0) and 0.25% deoxycholate) with protease inhibitors. After centrifugation at 12,000 rpm for 15 min at 4 °C, 1 mg of protein lysates were pre-cleaned with IgG for one hour, followed by incubation with the mouse anti-DYKDDDDK-Tag conjugated protein A/G beads (Abmart, M200018) overnight. Pre-cleaned brain lysates were incubated with primary antibodies or control IgG overnight, followed by incubation with protein A/G sepharose beads (Santa Cruz, sc2003) for one hour. The beads were then washed and immunoprecipitated proteins were eluted by boiling in loading buffer.

ChIP assays were performed with serum-shocked synchronized Neuro-2a cells as previously described [36]. Briefly, Neuro-2a cells cultured in 15-cm plates were washed twice by PBS and cross-linked with 1% formaldehyde for 15 min. Cross-linked cells were washed by ice-cold PBS and collected. The nuclear fractions were extracted by high-salt buffer followed by sonication three times for 10s at the maximum setting (SCIENTZ, Scientz-II D). The fragmentation of sonicated chromatin was evaluated by agarose gel electrophoresis and the sonication condition was optimized to achieve ideal fragment size of 200 – 1,000 bp. After centrifugation for 10 min at 12,000 rpm, the supernatants were immunocleared with 2μg sheared salmon sperm DNA (Sigma, D1626), IgG and protein A/G sepharose beads for 2 hrs at 4 °C, and immunoprecipitated with indicated antibodies overnight followed by incubation with salmon sperm DNA and protein A/G sepharose beads. Precipitates were de-crosslinked at 65 °C for 8 hrs and DNA was purified with Universal DNA Purification Kit (Tiangen, DP214) and used in quantitative PCR. For qPCR, 1.5 μL from 60 μL DNA extraction were used with specific primer pairs (Additional file 2: Table S1) and SYBR Green (BIO-RAD, 170888).

### Bioinformatics analysis

For Fig. 1d, *Fus* expression pattern in sleep deprived mouse brain was obtained from GSE9442 [31]. For Fig.1e and f, mouse liver and brain stem circadian microarray data were from GSE119237 [37] and GSE54650 [38] respectively. The circadian oscillation of gene expression was determined by fitting the circadian time-series data to cosine functions with 24 hours’ period and shifting phases as described previously [26]. For Fig. 2a, REV-ERBα and REV-ERBβ ChIP-seq data in mouse liver were mapped to mouse genome (mm9) by bowtie2 program [39] (default parameters). MACS program [40] was applied to identify the binding sites (MACS 1.4.2, default parameter) from ChIP-seq data. For Fig. 2f, BETA program [41] were applied to evaluate the activating/repressive function of REV-ERBα in mouse liver from the genome-wide data (with parameters:--df=0.05 -d 30000 -k BSF --da 500).

### Statistical analysis

Data are presented as mean ± s.e.m. and were analyzed by Prism 7 software (GraphPad). Two-tailed unpaired *t-*test was used to compare the means of two groups. One-way ANOVA followed by Newman-Keuls multiple comparisons test was used for multiple comparisons. Two-way ANOVA followed by Sidak multiple comparison test was used for analysis gene expression in various time points.

## Results

### FUS is a circadian clock-regulated gene

We first examined the protein expression of FUS in various mouse tissues, including the peripheral circadian clock center liver [42, 43], hypothalamus which harbors central circadian pacemaker suprachiasmatic nucleus[44], and cortex (Fig. 1a-c). FUS expression showed clear circadian oscillation although the patterns varied in different tissues. Since sleep deprivation (SD) affects the expressions of many core circadian genes [31, 45, 46], we compared the expression of *Fus* in the normal and SD conditions by a meta-analysis of published microarray datasets [31] (Fig. 1d). The basal level of *Fus* mRNA was decreased and oscillation amplitude was diminished in sleep-deprived mice (Fig. 1d). These results suggested that the expression of *Fus* was regulated by circadian clock. Furthermore, the oscillation of *Fus* expression was much stronger than *Tdp-43*, another ALS and FTD-associated nuclear protein [47–50] in mice, suggesting that FUS may exert a more active role in circadian rhythm (Fig. 1e, f).

To investigate the underlying mechanism of circadian regulation of *Fus*, we performed a bioinformatics analysis of published ChIP-seq datasets of core circadian proteins and found that only REV-ERBα, not even REV-ERBβ, bound within a 350 bp region in the *Fus* promoter [32], although the *Fus* promoter lacked a consensus REV-ERBα-binding site, the retinoic acid–related orphan receptor response (ROR) element (Fig. 2a, Additional file 3: Table S2). We confirmed the binding of REV-ERBα to the *Fus* promoter using chromatin immunoprecipitation (Fig. 2b). Silencing the gene encoding REV-ERBα, *Nr1d1*, reduced the expression from the *Fus* promoter, and deletion of the REV-ERBα-binding region relieved the effect of *Nr1d1* silencing (Fig. 2c). Intriguingly, removing the 350 bp region containing the REV-ERBα-binding site led to a REV-ERBα-independent increase of *Fus* promoter-luciferase activity, indicating that this region harbored a repressor-responsive element. Furthermore, the protein expression of FUS was reduced in *Nr1d1* knock-out MEF cells (Fig. 2d) as well as in the liver of *Nr1d1* knock-out mice (Fig. 2e), although the number of *Nr1d1* knock-out mice in our experiment was restricted by limited availability due to reduced fertility of these mice [5, 51]. Although REV-ERBα was usually reported as a transcriptional repressor [32], it regulated FUS as an activator in a way very similar to a *Drosophila Nr1d1* homolog, E75 [52], suggesting its versatile action depending on the presence of other transcriptional co-factors. To evaluate the global transcriptional regulation by REV-ERBα, we conducted an activating/repressive functional prediction analysis [41] based on the published REV-ERBα ChIP-seq data [32] and transcriptional profile from the *Nr1d1* knock-out mice [53], and found that REV-ERBα could activate a large number of genes it binds (Fig. 2f). These results indicate that *Fus* is a REV-ERBα-regulated circadian gene.

### FUS regulates the expression of core circadian genes

**Fig. 3:**
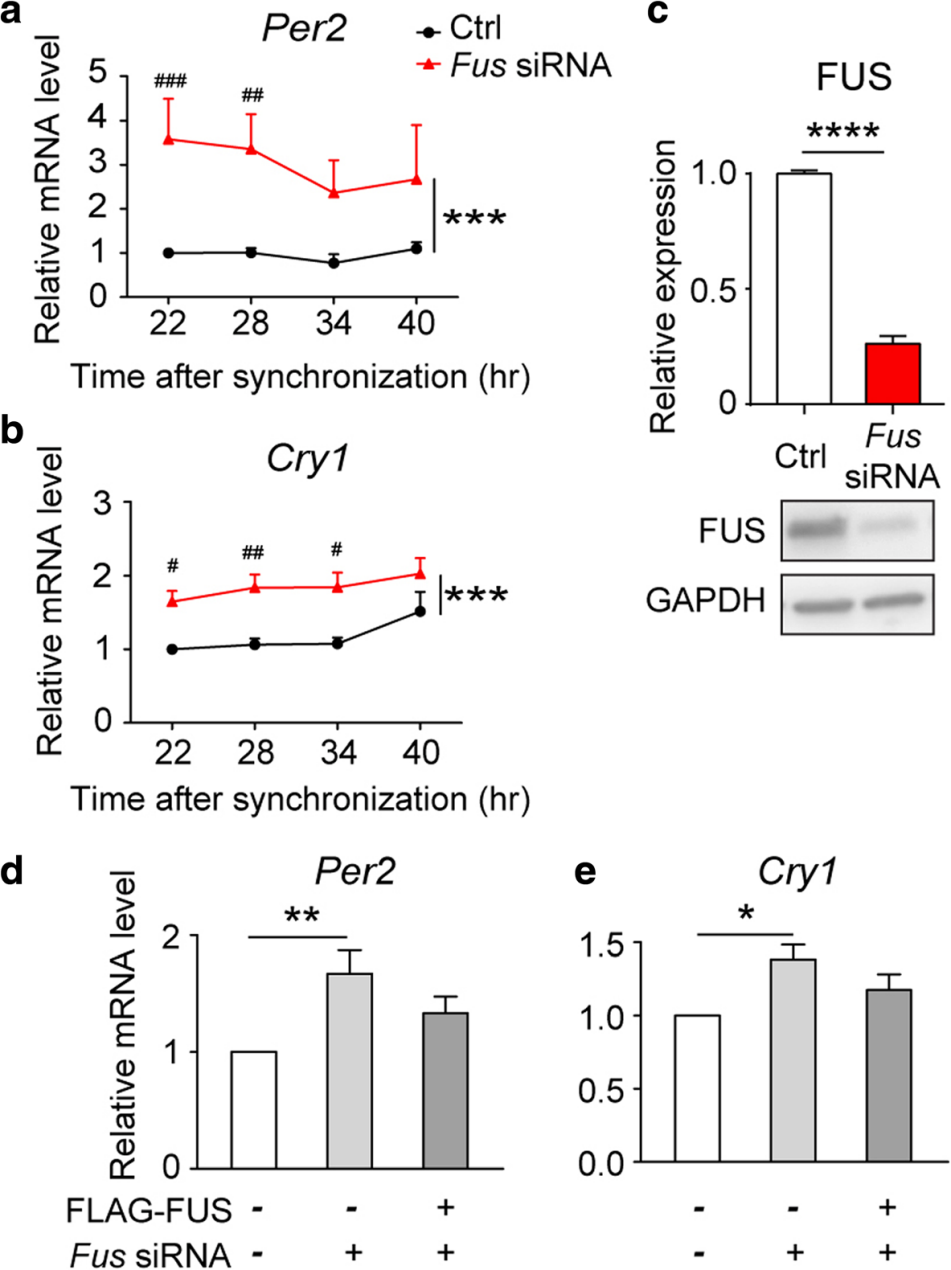
FUS regulates the expression of core circadian genes. **a**, **b** mRNA expression levels of *Per2* (**a**) and *Cry1* (**b**) in synchronized Neuro-2a cells after siRNA silencing (mean ± s.e.m.; *N* = 4-6 experiments; two-way ANOVA with Sidak’s multiple comparison test, * represents the significant *P-*value of overall two-way ANOVA analysis, ***:*P*≤0.001, # represents the significant *P-*value of Sidak’s multiple comparison test, #:*P*≤0.05, ##:*P*≤0.01, ###:*P*≤0.001). **c** Western blot showing the knock-down efficiency of *Fus*-targeting siRNA (mean ± s.e.m.; *N* = 3-4 experiments; *t*-test; ****: *P*≤0.0001). **d**, **e** RT-qPCR showing the mRNA expression of *Per2* and *Cry1* in cells with indicated transfection conditions in serum shock-synchronized Neuro-2a cells (mean ± s.e.m.; *N* = 8-9 experiments; One-way ANOVA with Newman-Keuls multiple comparisons test; *:*P*≤0.05; **:*P*≤0.01)

The diurnal expression of core circadian genes, such as *Per* and *Cry*, are regulated by complicated negative feedback loops involving transcriptional and post-translational regulation [1–3]. Since FUS is nuclear protein known to regulate gene expression [54], we assessed whether FUS may affect the expression patterns of some key genes in circadian control. We knocked-down the expression of endogenous *Fus* in synchronized Neuro-2a cells and found the mRNA levels of *Per2* and *Cry1* were generally increased with silenced expression of FUS at various time points during a diurnal cycle (Fig. 3a-c). Conversely, restoring the expression of FUS blunted the activation of *Per2* and *Cry1* by FUS depletion (Fig. 3d, e). These results suggest that FUS is a potential circadian regulator.

### FUS facilitates the recruitment of co-repressor complex to the promoters of *Per* genes

**Fig. 4:**
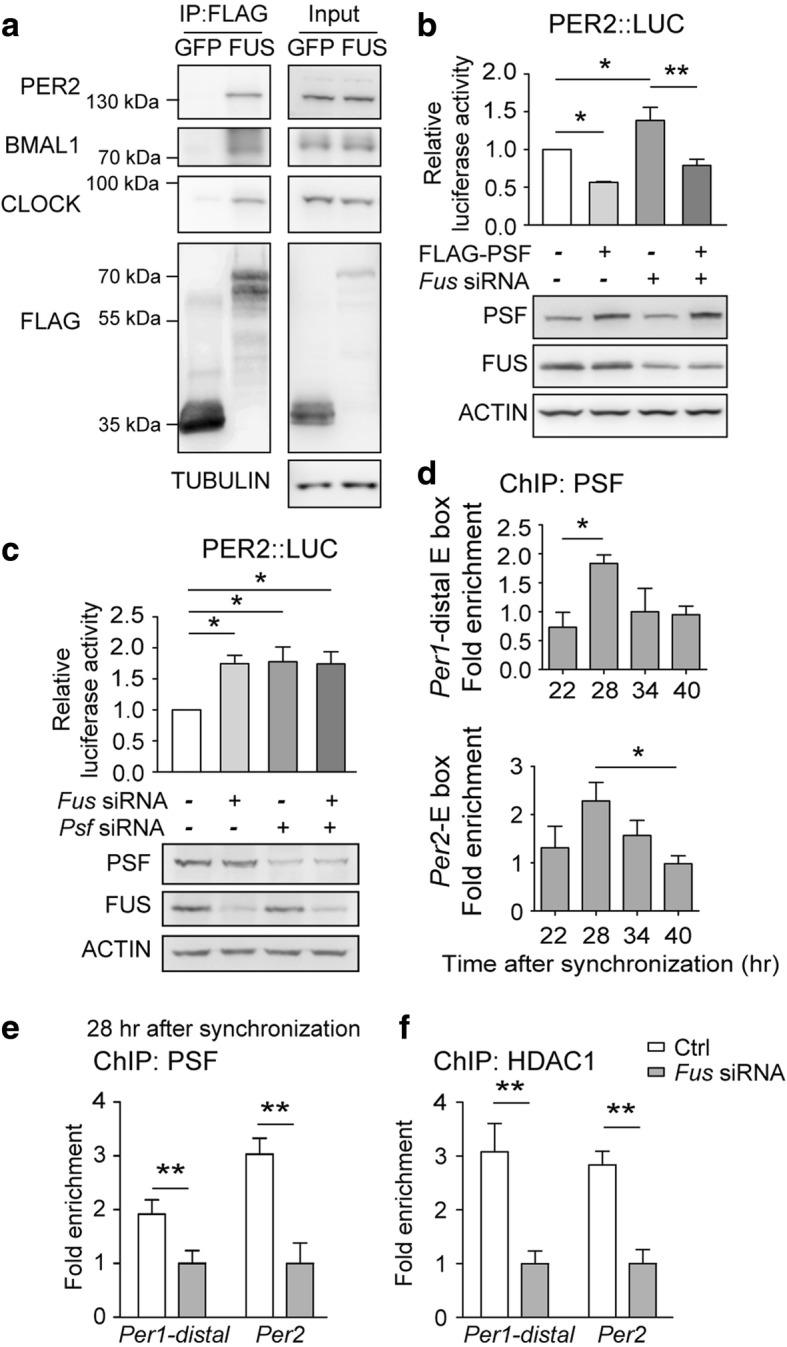
FUS is in the repressor complex and mediates the recruitment of PSF-HDAC1 to the promoters of E box-containing genes. **a** Co-immunoprecipitation of FLAG-FUS with PER2, BMAL1 and CLOCK in HEK293T cells. **b**, **c** *Per2* promoter-luciferase reporter activity in synchronized Neuro-2a cells after transfection and siRNA silencing with indicated conditions. Cells were first transfected with indicated RNAi constructs at 0 hr, followed by expression plasmid transfection at 24 hr if needed. Cells were synchronized at the 48 hr point for 2 hrs, and harvested for analysis at the 72 hr time point (mean ± s.e.m.; *N* = 3 experiments in duplicates; One-way ANOVA followed by Newman-Keuls multiple comparisons test; *:*P*≤0.05, **:*P*≤0.01). **d** ChIP-qPCR showing the promoter loading of PSF relative to IgG control. Neuro-2a cells were harvested at indicated time points after serum shock synchronization (mean ± s.e.m.; *N* = 3-5 experiments; One-way ANOVA followed by Dunnett's multiple comparison test; *:*P*≤0.05). **e**, **f** ChIP-qPCR showing the effect of FUS silencing on the binding of endogenous PSF (**e**) or HDAC1 (**f**) onto the E box-containing promoters of circadian genes. Neuro-2a cells were harvested at 28 hrs after serum shock synchronization (IgG as the ChIP control, mean ± s.e.m.; *N* = 3-5 experiments; *t*-test; **:*P*≤0.01)

We have previously shown that FUS interacts with PSF [35]. Interestingly, PSF is in the PER/CRY, BMAL1, CLOCK protein complex and negatively regulates the expression of *Per1* by recruiting the Sin3A-HDAC1 complex [12]. Using co-immunoprecipitation assay, we found that FUS could similarly bind PER2, BMAL1, and CLOCK (Fig. 4a). To evaluate the possibility that FUS and PSF may cooperatively repress *Per2* promoter-luciferase activity, we transfected PSF, in the presence or absence of FUS RNAi construct, and found that over-expression of PSF could reduce the *Per2* activation by FUS RNAi (Fig. 4b). Next, we found that while the knock-down of either FUS or PSF led to increased *Per2* promoter-luciferase activity, double knock-down did not produce an additive effect, suggesting that PSF and FUS operate through a common repressor complex (Fig. 4c). After analyzing the promoter loading of PSF at different circadian time in synchronized Neuro-2a cells, we found that during the observation window between 22 to 40 hrs after synchronization, the 28-hr time point showed the peak PSF binding (Fig. 4d). We then assessed the effect of FUS on the loading of PSF and HDAC1 onto the promoters of *Per1*, and *Per2* at this time point. FUS depletion led to reduced promoter occupancy of PSF and HDAC1 (Fig. 4e, f). Collectively, our results indicate that FUS may facilitate the recruitment of co-repressor complex PSF-HDAC1 to the promoters of *Per* genes.

### Mutation in *Fus* leads to abnormal circadian gene expression

**Fig. 5:**
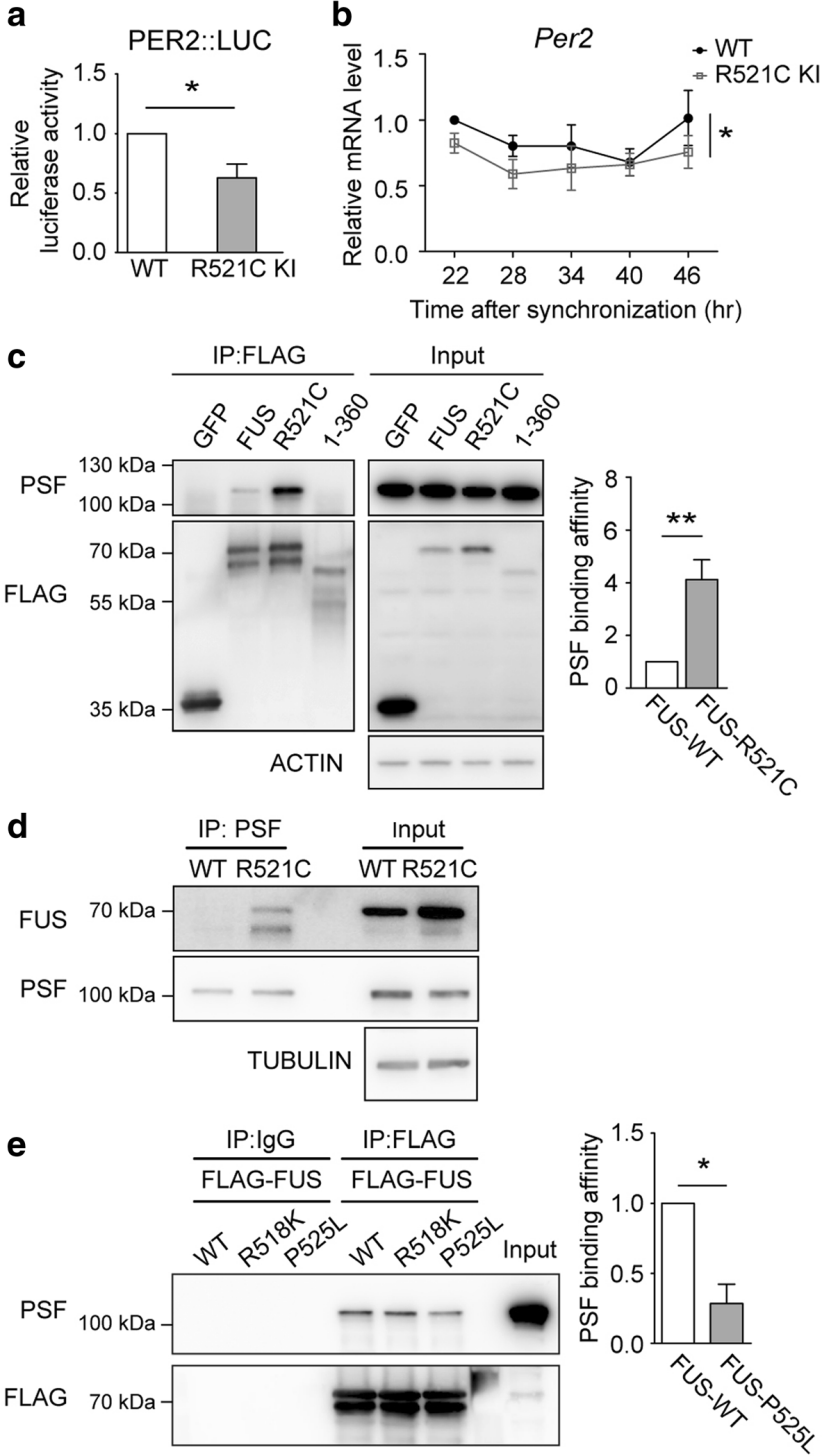
The pathogenic R521C mutation of FUS leads to abnormal expression of core circadian genes. **a** Luciferase activity of transfected PER2-promoter-luciferase in the wild-type (WT) and FUS R521C knock-in (KI) REFs (mean ± s.e.m.; *N* = 3 experiments; *t*-test; *:*P*≤0.05). **b** mRNA expression levels of *Per2* in synchronized REFs (mean ± s.e.m.; *N* = 6 experiments; two-way ANOVA, *:*P*≤0.05). **c** Co-immunoprecipitation of FLAG-GFP, wild-type or R521C-FUS and a FUS C-terminus deletion construct (1-360 residues) with endogenous PSF in HEK293T cells (mean ± s.e.m.; *N* = 5 experiments; *t*-test; **:*P*≤0.01). **d** Co-immunoprecipitation of endogenous PSF with FUS in the whole brain tissue of the wild-type and FUS R521C knocked-in rats. **e** Co-immunoprecipitation of FLAG-tagged wild-typed and R518K and P525L FUS with endogenous PSF in HEK293T cells (mean ± s.e.m.; *N* = 3 experiments, *t*-test, *:*P*≤0.05)

Mutations in *Fus* cause early onset of ALS and FTD [19–21, 55–57]. To evaluate whether pathogenic mutations may affect circadian regulation by FUS, we examined the expression of *Per2* and *Bmal1* in REFs derived from FUS R521C knock-in rats available in the lab. R521C is one of the most common FUS point mutations [58–61]. Both the *Per2* promoter activity and mRNA expression were decreased (Fig. 5a, b).We then assessed the interaction between PSF and FUS-R521C. We found that FUS-R521C showed a much stronger binding to PSF than the wild-type FUS and the binding between FUS and PSF required the C-terminus of FUS (Fig. 5c). Furthermore, we confirmed the increased binding between FUS R521C and PSF in the brain tissue from FUS-R521C knock-in rats (Fig. 5d). We have also detected the binding between PSF and other two C-terminus-located FUS pathogenic mutations, R518K and P525L (Fig. 5e). Surprisingly, unlike R521C, FUS P525L showed significantly weaker binding with PSF than wild-type FUS (more in Discussion). Taken together, our results suggest that mutations in *Fus* could lead to abnormal circadian gene expression.

## Discussion

In summary, we have identified ALS/FTD-associated FUS as a novel modulator for the circadian gene expression. The FUS expression is positively regulated by REV-ERBα. Meanwhile, FUS is a component of the PER-CRY-BMAL1-CLOCK mega-complex, and it represses the expression of *Per* by recruiting PSF-HDAC1. Therefore, besides transcriptionally repressing *Bmal1,* REV-ERBα could negatively regulate the expression of *Per* genes via the action of FUS.

The regulation of FUS by REV-ERBα suggests that dysregulated circadian clock could lead to the abnormal expression of FUS, as demonstrated by the reduction of FUS expression in sleep-deprived mice (Fig. 1d). Since the change in *Fus* expression is detrimental to neuronal health and contributes to the pathogenesis of ALS and FTD [58, 62, 63], our results have provided new mechanistic insights into the role of circadian dysfunction as a risk factor for neurodegeneration. Because of the importance of FUS, dramatic fluctuation of FUS would not be desirable for the organism. It is not surprising to observe the moderate but reproducible regulation of FUS by REV-ERBα. In addition, it is possible that other circadian regulators may also participate in the regulation of FUS expression.

Although we have described a mechanism related to the transcriptional regulation by FUS, other functions of FUS could also contribute to circadian gene regulation. For example, FUS is a key regulator in RNA splicing [64, 65]. However, published RNA splicing targets by FUS do not contain core circadian genes [64]. Whether the RNA-binding property of FUS is involved in circadian gene regulation remains to be elucidated.

Our data suggest that the FUS C-terminus, a mutation hot spot [66], may mediate the binding between FUS and the PSF-containing protein complex. Consistent with a recent report [67], pathogenic mutations such as R518K and P525L (Fig. 5e) could affect the binding between FUS and PSF. Therefore, some pathogenic mutations in *Fus* will conceivably lead to altered recruitment of the repressor complex and affect the transcription of core circadian genes. It will be of great interest in the future to determine whether FUS mutation-induced circadian gene dysregulation may contribute to or further exacerbate the neurodegeneration process.

## Conclusions

We have identified ALS/FTD-associated FUS as a modulator of circadian gene expression, and provided new mechanistic evidence supporting the mutual influence between circadian disturbance and neurodegeneration.

## Additional files

Additional file 1: **Figure S1.** The establishment of R521C FUS knock-in (KI) rats. (DOCX 92 kb)

Additional file 2: **Table S1.** Primer sequences. (DOCX 17 kb)

Additional file 3: **Table S2.** Predicted REV-ERBα-binding site on FUS promoter. (DOCX 14 kb)

## Abbreviations

*Per*: Period
*Cry*: Cryptochrome
*Nr1d1*: Nuclear receptor subfamily 1 group D member 1
PSF: PTB-associated splicing factor
HDAC1: Histone deacetylase 1
SIN3A: SIN3 transcription regulator family member A
GAPDH: Glyceraldehyde 3-phosphate dehydrogenase
NS: Non-significant
KO: Knock-out
KI: Knock-in
SD: sleep deprivation
CT: circadian time
RT-qPCR: Reverse transcription-quantitative polymerase chain reaction

## References

[1] Takahashi JS (2016). Transcriptional architecture of the mammalian circadian clock. Nat Rev Genet.

[2] Dibner C, Schibler U, Albrecht U (2010). The Mammalian Circadian Timing System: Organization and Coordination of Central and Peripheral Clocks. Ann Rev Physiol.

[3] Novak B, Tyson JJ (2008). Design principles of biochemical oscillators. Nat Rev Mol Cell Biol.

[4] Zhang Y, Fang B, Emmett MJ, Damle M, Sun Z, Feng D, Armour SM, Remsberg JR, Jager J, Soccio RE (2015). GENE REGULATION. Discrete functions of nuclear receptor Rev-erbalpha couple metabolism to the clock. Science.

[5] Preitner N, Damiola F, Lopez-Molina L, Zakany J, Duboule D, Albrecht U, Schibler U (2002). The orphan nuclear receptor REV-ERBalpha controls circadian transcription within the positive limb of the mammalian circadian oscillator. Cell.

[6] Gekakis N, Staknis D, Nguyen HB, Davis FC, Wilsbacher LD, King DP, Takahashi JS, Weitz CJ (1998). Role of the CLOCK protein in the mammalian circadian mechanism. Science.

[7] Kume K, Zylka MJ, Sriram S, Shearman LP, Weaver DR, Jin X, Maywood ES, Hastings MH, Reppert SM (1999). mCRY1 and mCRY2 are essential components of the negative limb of the circadian clock feedback loop. Cell.

[8] Shearman LP, Sriram S, Weaver DR, Maywood ES, Chaves I, Zheng B, Kume K, Lee CC, van der Horst GT, Hastings MH, Reppert SM (2000). Interacting molecular loops in the mammalian circadian clock. Science.

[9] Lee C, Etchegaray JP, Cagampang FR, Loudon AS, Reppert SM (2001). Posttranslational mechanisms regulate the mammalian circadian clock. Cell.

[10] Kim J, Kwak P, Weitz CJ (2014). Specificity in Circadian Clock Feedback from Targeted Reconstitution of the NuRD Corepressor. Molecular Cell.

[11] Padmanabhan K, Robles MS, Westerling T, Weitz CJ (2012). Feedback regulation of transcriptional termination by the mammalian circadian clock PERIOD complex. Science.

[12] Duong HA, Robles MS, Knutti D, Weitz CJ (2011). A molecular mechanism for circadian clock negative feedback. Science.

[13] Chang HC, Guarente L (2013). SIRT1 mediates central circadian control in the SCN by a mechanism that decays with aging. Cell.

[14] Asher G, Gatfield D, Stratmann M, Reinke H, Dibner C, Kreppel F, Mostoslavsky R, Alt FW, Schibler U (2008). SIRT1 regulates circadian clock gene expression through PER2 deacetylation. Cell.

[15] Musiek ES, Xiong DD, Holtzman DM (2015). Sleep, circadian rhythms, and the pathogenesis of Alzheimer disease. Exp Mol Med.

[16] Videnovic A, Lazar AS, Barker RA, Overeem S (2014). ‘The clocks that time us’--circadian rhythms in neurodegenerative disorders. Nat Rev Neurol.

[17] Lim C., Allada R. (2013). ATAXIN-2 Activates PERIOD Translation to Sustain Circadian Rhythms in Drosophila. Science.

[18] Zhang Y, Ling J, Yuan C, Dubruille R, Emery P (2013). A role for Drosophila ATX2 in activation of PER translation and circadian behavior. Science.

[19] Kwiatkowski TJ, Bosco DA, Leclerc AL, Tamrazian E, Vanderburg CR, Russ C, Davis A, Gilchrist J, Kasarskis EJ, Munsat T (2009). Mutations in the FUS/TLS gene on chromosome 16 cause familial amyotrophic lateral sclerosis. Science.

[20] Vance C, Rogelj B, Hortobagyi T, De Vos KJ, Nishimura AL, Sreedharan J, Hu X, Smith B, Ruddy D, Wright P (2009). Mutations in FUS, an RNA processing protein, cause familial amyotrophic lateral sclerosis type 6. Science.

[21] Deng H, Gao K, Jankovic J (2014). The role of FUS gene variants in neurodegenerative diseases. Nat Rev Neurol.

[22] Ahmed Rebekah M., Newcombe Rowena E.A., Piper Amanda J., Lewis Simon J., Yee Brendon J., Kiernan Matthew C., Grunstein Ron R. (2016). Sleep disorders and respiratory function in amyotrophic lateral sclerosis. Sleep Medicine Reviews.

[23] Bonakis Anastasios, Economou Nicholas-Tiberio, Paparrigopoulos Thomas, Bonanni Enrica, Maestri Michelangelo, Carnicelli Luca, Di Coscio Elisa, Ktonas Periklis, Vagiakis Emmanouil, Theodoropoulos Panagiotis, Papageorgiou Sokratis G. (2013). Sleep in Frontotemporal Dementia is Equally or Possibly More Disrupted, and at an Earlier Stage, When Compared to Sleep in Alzheimer's Disease. Journal of Alzheimer's Disease.

[24] Liguori Claudio, Placidi Fabio, Albanese Maria, Nuccetelli Marzia, Izzi Francesca, Marciani Maria Grazia, Mercuri Nicola Biagio, Bernardini Sergio, Romigi Andrea (2014). CSF beta-amyloid levels are altered in narcolepsy: a link with the inflammatory hypothesis?. Journal of Sleep Research.

[25] Kornmann B, Schaad O, Bujard H, Takahashi JS, Schibler U (2007). System-driven and oscillator-dependent circadian transcription in mice with a conditionally active liver clock. PLoS Biol.

[26] Yan J, Wang H, Liu Y, Shao C (2008). Analysis of Gene Regulatory Networks in the Mammalian Circadian Rhythm. PLoS Comput Biol.

[27] Bae S, Park J, Kim JS (2014). Cas-OFFinder: a fast and versatile algorithm that searches for potential off-target sites of Cas9 RNA-guided endonucleases. Bioinformatics.

[28] Yagita K, Okamura H (2000). Forskolin induces circadian gene expression of rPer1, rPer2 and dbp in mammalian rat-1 fibroblasts. FEBS Lett.

[29] Balsalobre A, Damiola F, Schibler U (1998). A serum shock induces circadian gene expression in mammalian tissue culture cells. Cell.

[30] Balsalobre A, Brown SA, Marcacci L, Tronche F, Kellendonk C, Reichardt HM, Schutz G, Schibler U (2000). Resetting of circadian time in peripheral tissues by glucocorticoid signaling. Science.

[31] Maret S, Dorsaz S, Gurcel L, Pradervand S, Petit B, Pfister C, Hagenbuchle O, O'Hara BF, Franken P, Tafti M (2007). Homer1a is a core brain molecular correlate of sleep loss. Proc Natl Acad Sci USA.

[32] Cho H, Zhao X, Hatori M, Yu RT, Barish GD, Lam MT, Chong LW, DiTacchio L, Atkins AR, Glass CK (2012). Regulation of circadian behaviour and metabolism by REV-ERB-alpha and REV-ERB-beta. Nature.

[33] Travnickova-Bendova Z, Cermakian N, Reppert SM, Sassone-Corsi P (2002). Bimodal regulation of mPeriod promoters by CREB-dependent signaling and CLOCK/BMAL1 activity. Proc Natl Acad Sci USA.

[34] Xu J, Kao SY, Lee FJ, Song W, Jin LW, Yankner BA (2002). Dopamine-dependent neurotoxicity of alpha-synuclein: a mechanism for selective neurodegeneration in Parkinson disease. Nat Med.

[35] Wang T, Jiang X, Chen G, Xu J (2015). Interaction of amyotrophic lateral sclerosis/frontotemporal lobar degeneration-associated fused-in-sarcoma with proteins involved in metabolic and protein degradation pathways. Neurobiol Aging.

[36] Shang Y, Hu X, DiRenzo J, Lazar MA, Brown M (2000). Cofactor dynamics and sufficiency in estrogen receptor-regulated transcription. Cell.

[37] Zhang R, Lahens NF, Ballance HI, Hughes ME, Hogenesch JB (2014). A circadian gene expression atlas in mammals: implications for biology and medicine. Proc Natl Acad Sci USA.

[38] Hughes ME, DiTacchio L, Hayes KR, Vollmers C, Pulivarthy S, Baggs JE, Panda S, Hogenesch JB (2009). Harmonics of circadian gene transcription in mammals. PLoS genetics.

[39] Langmead B, Salzberg SL (2012). Fast gapped-read alignment with Bowtie 2. Nature methods.

[40] Zhang Y, Liu T, Meyer CA, Eeckhoute J, Johnson DS, Bernstein BE, Nussbaum C, Myers RM, Brown M, Li W, Liu XS (2008). Model-based Analysis of ChIP-Seq (MACS). Genome Biol.

[41] Wang S, Sun HF, Ma J, Zang CZ, Wang CF, Wang J, Tang QZ, Meyer CA, Zhang Y, Liu XS (2013). Target analysis by integration of transcriptome and ChIP-seq data with BETA. Nat Protoc.

[42] Damiola F. (2000). Restricted feeding uncouples circadian oscillators in peripheral tissues from the central pacemaker in the suprachiasmatic nucleus. Genes & Development.

[43] Mohawk JA, Green CB, Takahashi JS (2012). Central and peripheral circadian clocks in mammals. Annu Rev Neurosci.

[44] Eastman CI, Mistlberger RE, Rechtschaffen A (1984). Suprachiasmatic nuclei lesions eliminate circadian temperature and sleep rhythms in the rat. Physiol Behav.

[45] Franken P, Thomason R, Heller HC, O’Hara BF (2007). A non-circadian role for clock-genes in sleep homeostasis: a strain comparison. BMC Neurosci.

[46] Wisor JP, O'Hara BF, Terao A, Selby CP, Kilduff TS, Sancar A, Edgar DM, Franken P (2002). A role for cryptochromes in sleep regulation. BMC Neurosci.

[47] Arai T, Hasegawa M, Akiyama H, Ikeda K, Nonaka T, Mori H, Mann D, Tsuchiya K, Yoshida M, Hashizume Y, Oda T (2006). TDP-43 is a component of ubiquitin-positive tau-negative inclusions in frontotemporal lobar degeneration and amyotrophic lateral sclerosis. Biochem Biophys Res Commun.

[48] Neumann M, Sampathu DM, Kwong LK, Truax AC, Micsenyi MC, Chou TT, Bruce J, Schuck T, Grossman M, Clark CM (2006). Ubiquitinated TDP-43 in frontotemporal lobar degeneration and amyotrophic lateral sclerosis. Science.

[49] Cairns NJ, Neumann M, Bigio EH, Holm IE, Troost D, Hatanpaa KJ, Foong C, White CL, Schneider JA, Kretzschmar HA (2007). TDP-43 in familial and sporadic frontotemporal lobar degeneration with ubiquitin inclusions. Am J Pathol.

[50] Mackenzie IR, Bigio EH, Ince PG, Geser F, Neumann M, Cairns NJ, Kwong LK, Forman MS, Ravits J, Stewart H (2007). Pathological TDP-43 distinguishes sporadic amyotrophic lateral sclerosis from amyotrophic lateral sclerosis with SOD1 mutations. Ann Neurol.

[51] Chomez P, Neveu I, Mansen A, Kiesler E, Larsson L, Vennstrom B, Arenas E (2000). Increased cell death and delayed development in the cerebellum of mice lacking the rev-erbA(alpha) orphan receptor. Development.

[52] Jaumouille E, Machado Almeida P, Stahli P, Koch R, Nagoshi E (2015). Transcriptional regulation via nuclear receptor crosstalk required for the Drosophila circadian clock. Curr Biol.

[53] Fang B, Everett LJ, Jager J, Briggs E, Armour SM, Feng D, Roy A, Gerhart-Hines Z, Sun Z, Lazar MA (2014). Circadian Enhancers Coordinate Multiple Phases of Rhythmic Gene Transcription In Vivo. Cell.

[54] Tan AY, Riley TR, Coady T, Bussemaker HJ, Manley JL (2012). TLS/FUS (translocated in liposarcoma/fused in sarcoma) regulates target gene transcription via single-stranded DNA response elements. Proc Natl Acad Sci U S A.

[55] Neumann M, Rademakers R, Roeber S, Baker M, Kretzschmar HA, Mackenzie IR (2009). A new subtype of frontotemporal lobar degeneration with FUS pathology. Brain.

[56] Seelaar H, Klijnsma KY, de Koning I, van der Lugt A, Chiu WZ, Azmani A, Rozemuller AJ, van Swieten JC (2010). Frequency of ubiquitin and FUS-positive, TDP-43-negative frontotemporal lobar degeneration. J Neurol.

[57] Urwin H, Josephs KA, Rohrer JD, Mackenzie IR, Neumann M, Authier A, Seelaar H, Van Swieten JC, Brown JM, Johannsen P (2010). FUS pathology defines the majority of tau- and TDP-43-negative frontotemporal lobar degeneration. Acta Neuropathol.

[58] Huang C, Zhou H, Tong J, Chen H, Liu Y-J, Wang D, Wei X, Xia X-G (2011). FUS Transgenic Rats Develop the Phenotypes of Amyotrophic Lateral Sclerosis and Frontotemporal Lobar Degeneration. PLoS Genetics.

[59] Sharma A, Lyashchenko AK, Lu L, Nasrabady SE, Elmaleh M, Mendelsohn M, Nemes A, Tapia JC, Mentis GZ, Shneider NA (2016). ALS-associated mutant FUS induces selective motor neuron degeneration through toxic gain of function. Nat Commun.

[60] Qiu H, Lee S, Shang Y, Wang WY, Au KF, Kamiya S, Barmada SJ, Finkbeiner S, Lui H, Carlton CE (2014). ALS-associated mutation FUS-R521C causes DNA damage and RNA splicing defects. J Clin Invest.

[61] Huang C, Tong J, Bi F, Wu Q, Huang B, Zhou H, Xia XG (2012). Entorhinal cortical neurons are the primary targets of FUS mislocalization and ubiquitin aggregation in FUS transgenic rats. Hum Mol Genet.

[62] Mitchell JC, McGoldrick P, Vance C, Hortobagyi T, Sreedharan J, Rogelj B, Tudor EL, Smith BN, Klasen C, Miller CC (2013). Overexpression of human wild-type FUS causes progressive motor neuron degeneration in an age- and dose-dependent fashion. Acta Neuropathol.

[63] Sabatelli M, Moncada A, Conte A, Lattante S, Marangi G, Luigetti M, Lucchini M, Mirabella M, Romano A, Del Grande A (2013). Mutations in the 3’ untranslated region of FUS causing FUS overexpression are associated with amyotrophic lateral sclerosis. Hum Mol Genet.

[64] Lagier-Tourenne C, Polymenidou M, Hutt KR, Vu AQ, Baughn M, Huelga SC, Clutario KM, Ling SC, Liang TY, Mazur C (2012). Divergent roles of ALS-linked proteins FUS/TLS and TDP-43 intersect in processing long pre-mRNAs. Nat Neurosci.

[65] Rogelj B, Easton LE, Bogu GK, Stanton LW, Rot G, Curk T, Zupan B, Sugimoto Y, Modic M, Haberman N (2012). Widespread binding of FUS along nascent RNA regulates alternative splicing in the brain. Sci Rep.

[66] Lattante S, Rouleau GA, Kabashi E (2013). TARDBP and FUS mutations associated with amyotrophic lateral sclerosis: summary and update. Hum Mutat.

[67] Ishigaki S, Fujioka Y, Okada Y, Riku Y, Udagawa T, Honda D, Yokoi S, Endo K, Ikenaka K, Takagi S (2017). Altered Tau Isoform Ratio Caused by Loss of FUS and SFPQ Function Leads to FTLD-like Phenotypes. Cell Rep.

