# Supplementary material for: Neurodegeneration-associated FUS is a novel regulator of circadian gene expression
Source: Transl Neurodegener. 2018 Oct 12;7:24. doi: 10.1186/s40035-018-0131-y (PMC6182827; doi:10.1186/s40035-018-0131-y)
Supplement: Supplementary file 1 — Figure S1. The establishment of R521C FUS knock-in (KI) rats. (DOCX 92 kb) [file 40035_2018_131_MOESM1_ESM.docx]

**Additional file 1**

**The establishment of R521C FUS knock-in (KI) rats**

(The following information was described in "**Sleep and circadian abnormalities precede cognitive deficits in R521C FUS knockin rats**", Tao Zhang et al, *Neurobiology of Aging*, 2018, in press)

The R521C FUS KI rats were generated by injecting Cas9 mRNA (100ng/μL), single guide RNA (sgRNA, 50ng/μL) targeting the C-terminus of rat *Fus* gene, and donor DNA (100ng/μL) into the cytoplasm of zygotes of the Sprague Dawley rats (Supplementary Figure 1). sgRNA sequence: TGAGCACAGACAGGATCGCA; donor DNA sequence: TTAATCTAACAAATAATTTTTTCTTTCAGGGGTGAGCACAGACAGG ATTGCAGGGAGAGGCCATATTAGCCTGACTCCTGAAGTTCTGGAACAGCTCTTC. The sgRNA and Cas9 mRNA were generated through PCR amplification with px330 plasmid [1] and transcription, and the donor DNA was artificially synthesized. Three hundred seventy-two injected embryos were transferred to 12 surrogates to obtain the initial 24 offsprings for genotype analysis. The presence of inserted mutation was determined by PCR followed by sequencing. The primers amplified from 267 bp upstream to 400 bp downstream of the *Fus* mutation site: 5’-TCCAGGTGAGTAAGTCAAAACA-3’; 3’-GAAACCACTCAAGGCAAACTAT-5’. Based on genotyping results of the F0 (founder) rats, two rats appeared to be heterozygous and two mosaic without any other mutation in the amplicon. One of the heterozygous F0 male rats (FUS/FUSR 521C) was used in subsequent breeding with the wild-type female rats. The potential off-target effects in F0 were estimated using Cas-OFFinder [2] and assessed by PCR-sequencing. To minimize the off target effects, we carried out six rounds of breeding. The elimination of off-target mutations in new generation rats used in various experiments was confirmed by PCR and sequencing.


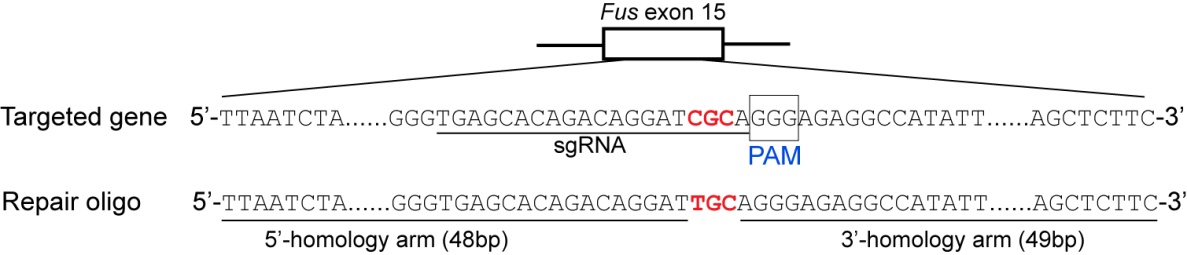


**Figure S1** Schematic representation of the homologous recombination-mediated T to C mutation at *Fus* R513 locus in rat genome by CRISPR/Cas9. A sgRNA was designed to bind the site coding the Arginine (R) at the 513 residue in FUS protein (equal to human FUS R521) and a double strand break was generated by RNA-guided DNA endonuclease enzyme Cas9 (CRISPR associated protein 9) near the binding site. Homologous recombination between FUS and a 100 bp repair oligo lead to a single nucleotide change from C to T (marked in red) and FUS Arginine 513 was mutated to FUS Cysteine (C) 513.

1. Cong L, Ran FA, Cox D, Lin S, Barretto R, Habib N, Hsu PD, Wu X, Jiang W, Marraffini LA, Zhang F: **Multiplex genome engineering using CRISPR/Cas systems.** *Science* 2013, **339:**819-823.

2. Bae S, Park J, Kim JS: **Cas-OFFinder: a fast and versatile algorithm that searches for potential off-target sites of Cas9 RNA-guided endonucleases.** *Bioinformatics* 2014, **30:**1473-1475.
