# Supplementary material for: Neurodegeneration-associated FUS is a novel regulator of circadian gene expression
Source: Transl Neurodegener. 2018 Oct 12;7:24. doi: 10.1186/s40035-018-0131-y (PMC6182827; doi:10.1186/s40035-018-0131-y)
Supplement: Supplementary file 2 — Table S1. Primer sequences. (DOCX 17 kb) [file 40035_2018_131_MOESM2_ESM.docx]

**Additional file 2**

**Table S1 Primer and siRNA sequences**

| **Primers for RT-qPCR** | | | |
| --- | --- | --- | --- |
| Mouse Actin-F | | GGCTACAGCTTCACCACCAC | |
| Mouse Actin-R | | GAGTACTTGCGCTCAGGAGG | |
| Mouse Nr1d1-F | | TGTCTCTGCAGACCGCTCG | |
| Mouse Nr1d1-R | | TTGCTTTTCCTTTTCGTCTCGT | |
| Mouse Cry1-F | | TTCCCTCCCTTGAAGCTCTC | |
| Mouse Cry1-R | | GAAGCAAAAATCGCCACCTG | |
| Mouse Per2-F | | GCGAAGCGCTTATTCCAGAG | |
| Mouse Per2-R | | AGTCTGAAGGCATCATCAGG | |
| Rat-Actin-F | | AGATCAAGATCATTGCTCCTCCT | |
| Rat-Actin-R | | ACGCAGCTCAGTAACAGTCC | |
| Rat-Per2-F | | GCTGCGAAGCGCCTCATTC | |
| Rat-Per2-R | | GGGGTGAGTGTTGGACGATT | |
| **Primers for ChIP-qPCR** | | | |
| Mouse Per1-distal E-box-F | | TCCGCAGTATTGGGTAAGTGTCGT | |
| Mouse Per1-distal E-box-R | | AAATCAGTGACGCAAATGCCAGCC | |
| Mouse Per2-1 E box-F | | AAGAGCGCGCAGCATCTTCATT | |
| Mouse Per2-1 E box-R | | ATTGGTCGGAGTGCCACCTCATTT | |
| Mouse Gapdh-F | | CATGGCCTTCCGTGTTCCTA | |
| Mouse Gapdh-R | | CCTGCTTCACCACCTTCTTGA | |
| Mouse Fus-in-(1-100)-F | | CCAGGCCTTGACTACACAGTT | |
| Mouse Fus-in-(1-100)-R | | CCTCTACCCCACTAGAGGCA | |
| Mouse Fus-in-(75-220)-F | | CCATTCTGCCTCTAGTGGGG | |
| Mouse Fus-in-(75-220)-R | | TACGGAGAGCTCAGGAAGACA | |
| **siRNAs** | | | |
| **Target** | **Sense (5’ – 3’)** | | **Antisense (5’ – 3’)** |
| Mouse Fus | CGUGGUGGCUUCAAUAAAUTT | | AUUUAUUGAAGCCACCACGTT |
| Mouse Nr1d1 | GGCUCAGCGUCAUAAUGAATT | | UUCAUUAUGACGCUGAGCCTT |
| Scrambled siRNA | UUCUCCGAACGUGUCACGUTT | | ACGUGACACGUUCGGAGAATT |
| siRNA targeting mouse PSF was purchased from QIAGEN (SI05783848) | | | |
