# Supplementary material for: Neurodegeneration-associated FUS is a novel regulator of circadian gene expression
Source: Transl Neurodegener. 2018 Oct 12;7:24. doi: 10.1186/s40035-018-0131-y (PMC6182827; doi:10.1186/s40035-018-0131-y)
Supplement: Supplementary file 3 — Table S2. Predicted REV-ERBα-binding site on FUS promoter. (DOCX 14 kb) [file 40035_2018_131_MOESM3_ESM.docx]

**Additional file 3**

**Table S2 Predicted REV-ERBα-binding site on FUS promoter**

CCAGGCCTTGACTACACAGTTCCGCTCTCGGGCGCAGGGCCCTGGCACCGCCCCTAGCGGGAGGCCGGGTCGTACCATTCTGCCTCTAGTGGGGTAGAGGTGTGTCGAAGGCGAGCCCCGGGCGACGGACGAGAGCCACCGGCCGCAAGGAGAAAGCGCGTGCGAGTGTTTGGGAATAAAGCGAGTTCGTTTTCCCTCTTGTCTTCCTGAGCTCTCCGTATCCCCGGGGAAGGGTGGGGACCTGGGCGTGCCTCCCCCTCGCGCTTCTCCACTTGGTACGTCCCGGTGGCGCGTAGGCGGCAGGCGTACTTAAGGCGGGCGCGGGAGGCGGGGCCGCTCAGTCCTCGCGG
